# Supplementary material for: Genomic Prediction Using Low Density Marker Panels in Aquaculture: Performance Across Species, Traits, and Genotyping Platforms
Source: Front Genet. 2020 Feb 27;11:124. doi: 10.3389/fgene.2020.00124 (PMC7056899; doi:10.3389/fgene.2020.00124)
Supplement: Supplementary Table 1 — Number of SNPs excluded because of different QC thresholds. [file Table_1.docx]

| **Species** | ***Cyprinus carpio*** | | ***Salmo salar*** | | ***Crassostrea gigas*** | | ***Sparus aurata*** | |
| --- | --- | --- | --- | --- | --- | --- | --- | --- |
| **Total SNPs** | **12311** |  | **16582** |  | **14058** |  | **12085** |  |
| MAF | -1308 |  | -5687 |  | 0 |  | -2533 |  |
| Marker call rate | -2500 |  | -224 |  | -30 |  | -1321 |  |
| HWE (pvalue 1e-6) | -1537 |  | -805 |  | 0 |  | -633 |  |
| **Passed filtering** | **6966** |  | **9866** |  | **14028** |  | **7598** |  |

**Supplementary Table 1.** Number of SNPs excluded because of different QC thresholds.
